# Occipital nerve stimulation for non-migrainous chronic headaches: a systematic review protocol

**DOI:** 10.1186/s13643-019-1101-x

**Published:** 2019-07-22

**Authors:** Sylvine Carrondo Cottin, Nevair Gallani, Léo Cantin, Michel Prud’Homme

**Affiliations:** 1 Neurosciences Unit, CHU de Québec–Université Laval Research Center, Québec, Québec Canada; 2 Instituto Neurologico de Sao Paulo, Functional Neurosurgery, Sao Paulo, Brazil; 3 Department of Surgery, Neurosurgery Division, CHU de Québec–Université Laval (Hôpital de l’Enfant-Jésus), Québec, Québec Canada; 4 Service de neurochirurgie, 1401 18E rue, Office D-3618, Québec, QC G1J 1Z4 Canada

**Keywords:** Occipital nerve stimulation, Pain relief, Chronic headache disorders

## Abstract

**Background:**

Defined as a headache lasting at least 15 days per month, chronic headache is reported by 3% of the general population, and a substantial proportion of them are refractory to current therapies. Occipital nerve stimulation (ONS) is a treatment option, but is still considered as a last resort treatment especially because of its invasive nature and the cost associated. Some reviews reported a limited efficacy of ONS for the treatment of migraines, with a high risk of complications. However, results reporting its efficacy and safety on other headache disorders are unclear. The aim of this review is to assess the efficacy and safety of ONS in regards to non-migrainous chronic headaches.

**Methods:**

We will conduct a systematic review and meta-analysis of studies evaluating the use of ONS in comparison to sham stimulation or the best available treatment in patients with chronic headache. MEDLINE, CINHAL, EMBASE, PsycINFO, ECRI Institute Library, WIKISTIM, the Cochrane Library databases, and clinical trial registries will be searched for eligible studies. The review will include adult patients diagnosed with chronic headache excluding migraine. Two independent reviewers will process to the screening of studies according to titles, abstracts, and then full texts. The primary outcome is the overall reduction of head pain severity. The secondary outcomes are rates of reduction in the severity of head pain, headache frequency, and duration, use of medication, impairment, quality of life, healthcare utilization, return to work, and adverse events. Extracted data will include patients’ and procedure characteristics, details on comparative treatment or sham, and clinical outcomes. The risk of bias of the studies will be also independently assessed using the Cochrane risk of bias tools.

**Discussion:**

This systematic review will allow us to better evaluate the potential role of ONS for the treatment of patients with chronic headache that are refractory to less invasive therapies. It will help to determine the degree of safety of ONS. Moreover, it will help to design and conduct future randomized controlled trials focused on patients who may better respond to such treatment.

**Systematic review registration:**

PROSPERO CRD42019121623

**Electronic supplementary material:**

The online version of this article (10.1186/s13643-019-1101-x) contains supplementary material, which is available to authorized users.

## Background

### Description of the condition

Headache disorders are a worldwide problem and are among the most common disorders of the nervous system affecting people of all ages, races, income levels, and geographical areas [1]. In 2011, the WHO has committed, with the non-governmental organization concerned by headache disorders, a global campaign to reduce the burden of headache [2]. With almost 3 billion individuals suffering from headaches all over the world, these disorders are responsible for a tremendous loss of economic resources all over the world [3]. Migraine, particularly, was estimated to have caused 45.1 million years of life lived with disability in 2016.

Although the most frequently studied, migraine is not the most common headache disorder. Tension-type headache and medication-overuse headache represent more than half of the prevalence of headache disorders [4] and are considered to be at least as costly as migraine for health-care systems [5].

Most of the people suffer at least once in their life from episodic headache, but when headache is persistent, it is qualified as chronic headache. Chronic headache, commonly defined as headache on 15 or more days every month for longer than 3 months, affects 1.7 to 4% of the adults worldwide [2]. Recognized as particularly debilitating, chronic headaches patients are often resistant or intolerant to the available treatment management [6].

### Description of the intervention

Occipital nerve stimulation (ONS) is a neurostimulation procedure consisting of subcutaneously implanting cylindrical or paddle leads over the occipital nerves in order to deliver electrical impulses aiming at alleviating pain [7]. The procedure is usually done in two stages, involving an initial trial of stimulation of a few days to a couple of weeks which, if successful, is followed by a permanent implant of a programmable pulse generator [8]. Some reviews reported a limited efficacy of ONS for the treatment of migraines, with a high risk of complications [9, 10]. However, results reporting its efficacy and safety on other headache disorders are unclear.

### How the intervention might work?

Cervical, somatic, and dural afferents have been shown to converge on second-order nociceptors in the trigeminocervical complex in animal studies [11, 12]. Moreover, suboccipital steroid injections have been shown to be effective for the prevention of several primary headaches [13, 14], supporting the rationale for an active role of occipital nerves in those disorders.

Various hypotheses have been emitted on the mechanism of action of ONS including a non-specific modulatory effect on pain-control systems [15] or normalization of the pain-matrix hyper-metabolism [16].

### Why it is important to do this review?

A guideline published by the National Institute for Health and Care Excellence in 2013 on ONS for chronic intractable migraine [17] recommends using this procedure with ‘special arrangements for clinical governance, consent, and audit or research.’ Indeed, ONS for intractable chronic migraine has shown some efficacy in the short term, but there is very little evidence about long-term outcomes. Moreover, there is a notable risk of complications, often needing further surgery that compromises the safety of this procedure [18]. The most frequent complications include lead migrations, lead erosions, infections, and lead fractures. The American Association of Neurological Surgeons (AANS) and Congress of Neurological Surgeons Joint Guideline Committees attributed a level III evidence for ONS in the treatment of occipital neuralgia [19]. On the European side, the European Headache Federation stated that the use of ONS seems acceptable for ‘the most severely affected patients with medically refractive chronic cluster headache’ [20]. Even if ONS for all indications is considered investigational and despite its ‘off-label’ status according to regulatory instances, ONS is becoming more and more used for treating various chronic headache disorders.

Considering the financial aspect, chronic migraines are responsible for total annual costs (including direct costs, i.e., medical treatment, and indirect costs, i.e., socioeconomic costs) of over $8000 in the USA [21]. Even if no cost-effectiveness data have been published about ONS, it could be expected that despite the high initial cost of this procedure, it would be cost-effective as compared to conventional medical management as it is for other invasive neuromodulation procedures [22].

Techniques and technology are rapidly evolving, and it becomes necessary to conduct well-designed studies able to assess the efficacy and safety of such devices.

## Objectives

The aim of this review is to evaluate the efficacy and safety of ONS with respect to the best available medical treatment or sham stimulation for patients suffering from chronic headaches excluding migraines.

## Methods

We propose to conduct a systematic review of every study reporting the use of occipital nerve stimulation in patients with chronic headaches.

### Protocol and registration

In accordance with the Preferred Reporting Items for Systematic Reviews and Meta-Analyses Protocols (PRISMA-P) 2015 statement [23], our systematic review protocol was registered with the International Prospective Register of Systematic Reviews (PROSPERO) [24] on January 2019 (registration number CRD42019121623).

### Study design

The review will be conducted and reported in compliance with the Preferred Reporting Items for Systematic Reviews and Meta-Analyses (PRISMA) statement [25] and The Cochrane Handbook for Systematic Reviews of Interventions [26] methodological recommendations.

### Eligibility criteria

Since we are interested in the safety and efficacy of ONS, our systematic review will include every study irrespective of their design. Studies on patients suffering from chronic headache (any etiology excluding migraine) according to The International Classification of Headache Disorders [27] will be considered and reported results mainly focused on chronic migraine where other types of headache disorders were also included will be considered. No restrictions will be applied neither on the hospital type (private or public), nor on the device used for neurostimulation, but studies that focused on the combined use of ONS with other forms of nerve stimulation will be excluded. Trials investigating the effect of stimulation of occipital nerves or areas innervated by them will be eligible. The comparator/control will include placebo/sham control or the best available treatment including injections, ablative techniques, and pharmacological or psychological interventions. Pre-post studies with internal comparison groups will also be included. Case reports, case series, in-progress clinical studies, and letters will be included in a qualitative analysis. Blinded as well as unblinded studies will be considered for inclusion in this review. Tables 1 and 2 present the structured study question and inclusion and exclusion criteria, respectively.

**Table 1:** Structured question

Population	-Adult patients with chronic headache
Intervention	-Occipital nerve stimulation
Comparator	-Any comparator
Primary outcome	-Pain relief
Secondary outcomes	-Headache frequency, intensity, and duration -Functional status -Quality of life -Return to work -Medication use -Healthcare utilization -Complications
Study designs	-Any observational or intervention design

**Table 2:** Study eligibility criteria

Inclusion criteria	-Randomized controlled trials, quasi-randomized trials, retrospective, and prospective observational studies -Chronic setting -At least one group of patients suffering from chronic headache -At least one group of adult patients (≥ 18 years old) -Any sample sizes -Clinical trials with at least one group of patients treated by occipital nerve stimulation -Studies including before and after internal control or a separate control group
Exclusion criteria	-Sample of patients with migraine only -Combination of ONS and other forms of nerve stimulation

### Information sources

We will systematically search MEDLINE (PubMed), Embase, CINHAL, PsycINFO, ECRI Institute Library, and The Cochrane Library databases (from their inception up to a maximum of 6 months before submission for publication) for relevant citations of published trials. The reference lists of narrative and systematic reviews and included trials will be hand-searched for relevant citations. Additionally, the main neuro-functional surgery practitioners worldwide will be contacted for information about publications that might not be identified using the search strategy. Companies that sell peripheral nerve stimulation devices will be enquired for relevant published or unpublished trial data, and studies highlighted during NICE’s public consultation of relevant *Interventional Procedures Guidance* will also be examined. Finally, clinical trial registries (Clinicaltrials.gov, WHO International Clinical Trials Registry Platform, ISRCTN registry, Cochrane Controlled Register of Trials (CENTRAL) and Health Canada’s Clinical Trials Database) will be searched and authors of pertinent trials will be contacted concerning upcoming publications.

### Search strategy

Searches will be conducted using index terms and keywords relating to chronic headache, as well as occipital nerve stimulation. Clinicians, investigators with expertise in functional neurosurgery and headaches, and information specialists will be consulted to verify the search strategy, identify synonyms and additional search terms. Relevant index terms (Medical Subject Headings and Emtree) will be added to the strategy. The search will be limited to human studies [26]. No language or date of publication restriction will be used. The search strategy will be first designed for MEDLINE and EMBASE, and will be adapted for other electronic databases afterward. The most recent version of our MEDLINE search strategy is presented in Additional file 1. This preliminary strategy will be tested through an iterative process in order to achieve sufficient specificity while maintaining high sensitivity. References will be managed in EndNote (version X8.2, New York City: Thomson Reuters, 2011) and duplicates will be removed. References will then be exported to a Microsoft Excel (version 16.16.11, Redmond, WA: Microsoft, 2018) spreadsheet in order to complete the selection process.

### Study selection

A two-stage process will be used for study screening and selection using standardized and pilot-tested screening forms. First, two independent reviewers will screen titles and abstracts of retrieved articles to determine whether a citation met the inclusion criteria. Then, if agreement on first stage selection is reached, reviewers will proceed to the full-text review of potentially eligible studies according to the predefined inclusion and exclusion criteria. Reasons for exclusions will be noted all along the screening process. In case of disagreement on the inclusion of a study, discrepancies will be resolved through a consensus obtained by a discussion with a third reviewer.

Agreement on study selection will be evaluated with a kappa coefficient (thresholds of 0.61 to 0.80 indicating substantial agreement and ≥ 0.81 indicating nearly perfect agreement) [28] and 95% confidence interval. In case the agreement is too low, indicating an evasive interpretation of eligibility criteria, a third reviewer will review records’ titles and abstracts. A translation will be performed for articles published in languages other than English, French, or Portuguese.

The elimination process for the analysis of studies will be shown using a flow diagram following the PRISMA model [25].

### Data collection process

Two independent reviewers will abstract data from included citations using a standardized form. This form will be pilot-tested and customized by two reviewers using a sample of selected publications. A percentage of agreement higher than 95% will be needed to consider the form adequate to proceed to a large-scale extraction. Would that requirement not satisfied, an agreement between reviewers will be reached on modifications to make. In case of discrepancy, consensus will be reached with the involvement of a third reviewer. Corresponding authors will be contacted if additional data are needed.

### Data items

Extracted data will include study characteristics (year of publication, design, language, study period, funding sources, location, study sample size, eligibility criteria for entering the study, blindness); patients’ characteristics (gender, age, and other relevant demographic data, type of headache and duration of symptoms, follow-up period); surgical procedure information (types of leads and implantable pulse generator, modality of implantation, stimulation parameters); and comparator details (details about any placebo or sham procedure performed, and of other treatments). The primary outcome will be the overall reduction of head pain severity (any scale and any treatment duration). Rates of reduction in the severity of head pain, headache frequency and duration, medication use, impairment, quality of life, healthcare utilization, and return to work will also be extracted as secondary outcomes. For the safety profile, we will consider the occurrence of post-surgery complications (infection, skin erosion, allergic reaction, hematoma, etc.), hardware-related complications (lead migration/dislodgement, battery failure, disconnection), and stimulation-related complications (discomfort, muscle spasms/cramping, nausea/vomiting). Details about any conflict of interest declared by the authors of the selected studies will be reported. In case of missing data, the reviewers will contact the authors.

### Risk of bias in individual studies

To determine the methodological quality of included RCTs, the risk of bias will be assessed independently by two reviewers for each individual study using The Revised Cochrane Risk of bias tool for randomized trials (RoB 2) [29]. For non-randomized studies, the risk of bias will be evaluated using the Cochrane tool Risk of Bias in Non-Randomized Studies of interventions (ROBINS-1) [30]. The quality of the trials will be considered in subgroup analyses.

### Synthesis of the results

Meta-analyses of risk ratios (for dichotomous outcomes) will be carried out in Review Manager 5.3.5 (Copenhagen: The Nordic Cochrane Centre, The Cochrane Collaboration, 2014) using Mantel-Haenszel random-effect models. Pooled effect sizes and their 95% confidence limits will be reported. Means and mean differences (for continuous outcomes) will be analyzed using the inverse variance method with random effects models. Statistical heterogeneity between studies will be measured using the Cochrane’s *Q* test and *I*^2^ statistics [31], the latter being interpreted as low from 0 to 40%, moderate from 30 to 60%, substantial from 50 to 90%, and considerable from 75 to 100% according to the Cochrane Handbook for Systematic Reviews of Interventions [26]. If sufficient studies are included in the meta-analysis, we plan to pursue sensitivity analyses based on study designs (including only randomized controlled trials or only studies with at least one external comparator group), and on population (including only studies with at least 50% of adults or 50% of patients suffering from chronic non-migrainous chronic headache).

### Reporting biases

Funnel plots will be created in order to evaluate the risk of publication bias by visual exploration. The risk of selective reporting of outcomes within studies will also be evaluated by searching for previously published protocols on registration website (clinicaltrials.gov, International Clinical Trials Registry Platform of the WHO, ISRCTN registry, Cochrane Controlled Register of Trials (CENTRAL), and Health Canada’s Clinical Trials Database).

### Additional analyses

#### Subgroup analyses

To assess the strength of observed associations and control for between studies heterogeneity, we will perform subgroup analyses according to headache etiology, type of leads, reported funding, symptoms duration, and risk of bias (low vs high risk of bias).

#### GRADE of evidences

We will use the GRADE methodology to evaluate the quality of evidences (www.gradeworkinggroup.org) of our findings according to four categories (high, moderate, low, or very low) [32].

## Discussion

### Expected benefits

This project will allow the knowledge synthesis regarding occipital nerve stimulation in adult patients with non-migrainous chronic headaches. Considering the paucity of rigorous data on the efficacy of this neurostimulation-based therapy in this population, it is of major importance to assess current practices.

### Inform future studies

Our results will provide information to advise the design of further studies in peripheral nerve stimulation and headache disorders. For example, information on the headache etiology for which ONS is the best effect will facilitate the practitioner in selecting patients who are predicted to better respond to this therapy.

### Limitations

Despite the use of rigorous and validated methodology, we do expect the retrieval of a limited number of randomized controlled trials including a small number of patients. This may prevent the planned subgroup analyses.

In addition, as this field concerns high technology devices, it is expected that most of the trials published were carried out or funded by companies manufacturing those devices. This may lead to unreliable conclusions.

## Additional file

Additional file 1: Search strategy. (DOCX 89 kb)

## Data Availability

The datasets used and/or analyzed during the current study are available from the corresponding author on reasonable request.

## Abbreviations

GRADE: Grading of Recommendations Assessment Development and Evaluation
ONS: Occipital Nerve Stimulation
PRISMA: Preferred Reporting Items for Systematic Reviews and Meta-Analyses
RCT: Randomized controlled trial
