# Supplementary material for: Occipital nerve stimulation for non-migrainous chronic headaches: a systematic review protocol
Source: Syst Rev. 2019 Jul 22;8:181. doi: 10.1186/s13643-019-1101-x (PMC6647252; doi:10.1186/s13643-019-1101-x)
Supplement: Supplementary file 1 — Search strategy. (DOCX 89 kb) [file 13643_2019_1101_MOESM1_ESM.docx]

**Search strategy for Medline (Pubmed)**

**Patients**

#1

(Headache [MeSH terms] OR "Cluster headache" [MeSH Terms] OR "Headache disorders" [MeSH])

#2

(Headache* [tiab] OR "Head Pain*" [tiab] OR "Cranial Pain*" [tiab] OR Cephalalgia* [tiab] OR Cephalgia* [tiab] OR Hemicrania [tiab] OR "Ciliary Neuralgia*" [tiab] OR "Horton* Syndrome" [tiab] OR "Bing Horton Syndrome" [tiab] OR "Occipital neuralgia*" [tiab] OR "Arnold neuralgia*" [tiab] OR "Arnold’s neuralgia" [tiab] OR "Arnold chiari neuralgia" [tiab] OR "Glossopharyngeal neuralgia*" [tiab] OR "Trigeminal neuralgia*" [tiab] OR "Painful Cranial Neuropath*" [tiab] OR "Trigeminal Neuropath*" [tiab] OR "Tolosa-Hunt Syndrome" [tiab] OR "Paratrigeminal oculosympathetic syndrome" [tiab] OR "Reader’s Syndrome" [tiab] OR "Reader Syndrome" [tiab])

#3

("Electric stimulation therapy" [MeSH] OR "Electric* Stimulation Therapy" [tiab] OR "Therapeutic Electric* Stimulation" [tiab] OR Electrotherapy [tiab] OR "Interferential Current Electrotherapy" [tiab] OR "Occipital nerve stimulat*" [tiab] OR "Occipital nerve neurostimulat*" [tiab] OR "Occipital nerve" [tiab] OR "Occipital neurostimulat*" [tiab] OR "Peripheral nerve stimulation" [tiab] OR "Peripheral nerve field stimulation" [tiab] OR Neurostimulation [tiab] OR Neuromodulation [tiab] OR ONS [tiab])

#4 : #1 OR #2 AND #3
